# The role of perceived organisational justice in the experience of pain among male and female employees

**DOI:** 10.1177/1359105320967423

**Published:** 2020-10-26

**Authors:** Joanna L McParland, Anne Gasteen, Martijn Steultjens

**Affiliations:** Glasgow Caledonian University, UK

**Keywords:** chronic pain, employees, organisational justice, sex differences, workplace

## Abstract

This study examined the association of organisational justice with pain among employees of a large organisation. Employees (*n* = 1829) completed measures of pain, fair pay, organisational justice, job satisfaction and stress. Logistic regression analyses found that organisational justice was unrelated to pain among women, but men with higher perceptions of fair pay were more likely to report chronic pain as were men with lower perceptions of distributive justice. This is the first study indicating that fair pay and distributive justice are both unique predictors of chronic pain in men. The findings have implications for supporting employees with chronic pain.

## Introduction

Pain is a major public health issue that is a burden on health and social care systems across the world. Chronic primary pain, defined as pain in at least one anatomical region that persists or recurs for longer than 3 months (Nicholas et al., 2019) affects up to half of the UK population (Fayaz et al., 2016), a pattern that is mimicked globally. Moderate or severe chronic pain can have a significant impact on an individual’s daily activities, including their working lives (Breivik et al., 2013). Chronic pain is a significant burden within the workplace, due to its effect on sickness absence, employment status and productivity (Patel et al., 2012; Philips, 2009).

While some people with chronic pain cannot work, many employees remain at work with pain (Pain Alliance Europe, 2018), although they may require workplace accommodations and have poorer wellbeing and less functional capacity to complete tasks than employees without pain (Rodrigues et al., 2017; Soer et al., 2012). Pain can be affected not only by the physical work environment but also by other, psychosocial work factors. This is consistent with the biopsychosocial formulation of pain which suggests that psychosocial factors can contribute to pain and suffering (Gatchel et al., 2007). Psychosocial factors are a central part of the work experience. They involve the workplace characteristics individuals are exposed to at work as well as employee beliefs about the workplace and are known risk factors for poor health and sickness absence (White et al., 2013). Much research has found numerous psychosocial work factors and work attitudes to be associated with chronic pain, including work demands, job control and social support (Buruck et al., 2019; Lang et al., 2012).

One specific work attitude that has implication for health and wellbeing and is a determinant of other workplace factors, is organisational justice (Van Dijke and De Cremer, 2016). Organisational justice is characterised by perceived fairness in relation to different work domains, including interpersonal treatment (e.g. dignity, respect), the distribution of outputs such as rewards and benefits and procedures for making decisions about the allocation of outputs. These types of organisational justice have had differential effects on the physical and mental health of employees (Ndjaboue et al., 2012; Robbins et al., 2012) with repercussions for sickness absence (Tenhiälä et al., 2013). Explanations for this association consider organisational justice as a stressor that adversely affects health through psychophysiological and/or behavioural mechanisms (Kompier and Taris, 2011; Robbins et al., 2012), although alternative plausible explanations suggest health status can affect perceptions of injustice because they triggers negative work attitudes and/or behaviour that elicits a change in the work environment (Eib et al., 2018).

Despite evidence of a clear association between organisational justice and health, relatively little is known about the role of organisational justice in the experience of pain. A small number of studies have reported a negative association between perceived organisational justice and pain (Freimann et al., 2016; Heponiemi et al., 2013; Herr et al., 2015; Manville et al., 2016; Pekkarinen et al., 2013; Saastamoinen et al., 2009), but beyond this virtually nothing is known about injustice in the context of pain and work, particularly which type or types of organisational justice are most relevant to the pain experience.

The aim of the present study was to examine the association of procedural, interpersonal and distributive justice with acute and chronic pain among male and female employees, compared to employees without pain. Fair pay is a component of distributive justice but was distinguished from this as distributive justice encompasses broader outcomes such as promotion, recognition and access to physical facilities, and we sought to examine the association of fair pay as a unique component of the workplace experience. Since organisational justice is part of a complex psychosocial work environment with direct implications for work stress (Matta et al., 2017) and job satisfaction (Herr et al., 2020) that are both risk factors for pain (Ariens et al., 2001; Bonzini et al., 2015), we included these variables to investigate the associations between each type of organisational justice and acute and chronic pain independently of these other important aspects of the psychosocial work environment.

## Materials and methods

### Study setting and participants

This study is part of a larger employee wellbeing study examining the health and pain status of employees of a large public sector organisation in Scotland. Ethical approval for the study was granted by the Department of Psychology, Social Work and Allied Health Sciences at Glasgow Caledonian University. Employees were emailed a web link to the study questionnaire by their employer through their work email address and hard copies were distributed within the organisation. Written or online consent was obtained prior to data collection. The total number of respondents was 1829 (approximate 10% response rate).

### Self-report measures

*Pain* Consistent with previous work (Elliot et al., 2014) pain was measured by asking participants whether they were currently troubled by pain or discomfort, either all the time, or on and off (Yes/No). Those who responded ‘Yes’ were asked whether they had experienced pain or discomfort for more than 3 months (Yes/No). Those who reported currently experiencing pain but not for 3 months or longer were categorised as having acute pain, while those who reported experiencing pain for at least three months were categorised as having chronic pain (International Association for the Study of Pain, 1986). All others were categorised as having no pain.

*Organisational justice* was measured using single item measures based on a modified version of items developed previously (Jordan and Turner, 2008). Distributive justice was measured with: ‘I feel the rewards I receive from working for my employer are fair. Pay, recognition and physical facilities are examples of rewards’. Procedural justice was measured with: ‘I feel the formal policies and procedures used by my employer to make decisions are fair’. Interpersonal justice was measured with: ‘In all aspects of my work environment, I feel that my primary supervisor treats me in a fair manner’. Fair pay was measured with: ‘I feel the pay I receive from working for my employer is fair’. Each item was measured on a 1–7 scale, from 1 (Strongly disagree) to 7 (Strongly agree). The reliability and concurrent validity of the scales has been established (Jordan and Turner, 2008).

*Job satisfaction* was measured using the four-item job satisfaction scale from the Copenhagen Psychosocial Questionnaire (COPSOQ) (version 2), a measure of the psychosocial work environment (Pejtersen et al., 2010). The scale measures satisfaction with work prospects, physical working conditions, the way one’s abilities are used and general job satisfaction. Each item was scored on a scale with the response options: ‘Very unsatisfied’, ‘Unsatisfied’, ‘Satisfied’ and ‘Very satisfied’. The optional fifth option, ‘Not relevant’ was excluded. Each item was scored on a 0–100 scale (i.e. 100, 66.7, 33.3 and 0, for a four-item category scale). If fewer than half of the scale questions were answered, then the scale score was set to missing. A total score was created as an average of the individual scores. A higher score indicated greater job satisfaction. The COPSOQ has been widely used and the scales have been found to be reliable and valid (Bjorner and Pejtersen, 2010; Thorsen and Bjorner, 2010). Within the present study, the internal consistency of the scale was 0.80 (Cronbach’s Alpha).

*Job stress* was measured using the four-item job stress scale from the COPSOQ (Version 2) (Pejtersen et al., 2010). The scale items measure stress in the past 4 weeks, in relation to the frequency of having a problem relaxing, irritability, tension and general stress. Each question was scored on a scale with the response options: ‘All the time’, ‘A large part of the time’, ‘Part of the time’, ‘A small part of the time’ and ‘Not at all’. Each item was scored on a 0–100 scale (i.e. 100, 75, 50, 25, 0 for a five-item response category scale). If fewer than half of the questions in the scale were answered, then the scale score was set to missing. A total score was created as an average of the individual scores. A higher score indicated greater stress. Within the present study, the internal consistency of the scale was 0.92 (Cronbach’s Alpha).

## Data sharing statement

The statistical data file corresponding to our analysis is available from the first author on request.

## Data analysis

Descriptive statistics for the sample were reported. Bivariate correlations to test for the presence of multicollinearity between organisational justice, psychological stress and job satisfaction variables produced correlations ranging in value from ‒0.19 to 0.69. The strongest correlation was between fair pay and distributive justice. Colquitt and Shaw (2005) indicate that correlations between organisational justice variables below 0.70 can be tolerated. Therefore, these variables were not aggregated.

Logistic regression analyses were conducted to examine the associations between these variables and each of three groups (no pain, acute pain, chronic pain), with ‘no pain’ as the reference group in the analysis. Logistic regression models relate the log odds of a binary outcome measure to explanatory variables, which may be categorical or continuous. In our analyses, 0 indicates the ‘no pain’ reference category while 1 indicates either acute or chronic pain. Logistic regression coefficients give the change in the log odds or percentage change in the odds for a successful outcome (i.e. that the dependent variable equals 1) associated with a unit change in an independent variable. Job-related, demographic and health-related variables were controlled for in the analyses. These variables included UK Standard Occupational Classifications (Professional, Associate Professional or Technical staff, Administrative and Secretarial staff), gross weekly earnings, employment status (full-time, part-time), age (categories 35–44, 45+, compared to 16–34 category), marital status (Cohabiting partner/No current partner), highest educational qualification (post-graduate, degree, Further Education or Highers, compared to low qualifications), self-reported general health (0–100, higher scores indicate better health) (Bowling, 2005), whether currently a cigarette smoker (Yes/No), the number of days per month a minimum of 30 minutes exercise has been completed (1–11 days, 12–19 days, 20+ days) (Milton et al., 2011) and total frequency and amount of alcohol consumed (scored 0–12; 5+ indicates higher risk drinking). Alcohol consumption was modelled in quadratic form (with a squared term) to reflect the likely non-linear relationship between chronic pain and alcohol consumption found within the literature (Skillgate et al., 2009). The organisational justice, job satisfaction and psychological stress variables were adjusted for confounders and simultaneously for each other (fully adjusted model). The results of the logistic regression are presented as odds ratios (OR) with their 95% confidence intervals (CI). Pseudo R^2^ values were included to provide an approximation of goodness of fit for the regression models. The tables report the regression models for pain for all the sample, and then separately for males and females.

## Results

### Descriptive statistics

Table 1 shows the descriptive statistics of the sample. Most of the sample was female (66%), in the age range 35–44 (45%) and either married or living with a partner (69%). Most employees had Further Education (29%) or Degree/Postgraduate qualifications (48%) and were employed full-time (88%), mainly in Administrative/Secretarial (32%), Associate Professional and Technical (29%) and Professional (23%) occupations. Of the 48% of employees reporting pain, most experienced chronic pain (40%), 70% of whom were female. Of those reporting chronic pain, 92% had at least one musculoskeletal pain condition, commonly back pain, joint pain and/or neck pain, or a musculoskeletal condition accompanied by another type of pain condition (headache, toothache, abdominal pain). The remaining 8% of those with chronic pain reported one or more of these other pain conditions.

**Table 1.** Sample characteristics.

	All *N* (%)	Men *N* (%)	Women *N* (%)
Respondents		625 (34)	1198 (66)
Age group (years)			
16–24	46 (2)	14 (2.3)	32 (3)
25–34	223 (12)	69 (11)	153 (13)
35–44	815 (45)	268 (43)	541 (45)
45–54	654 (36)	237 (38)	413 (34.5)
55+	91 (5)	33 (5.3)	58 (5)
Total	1829	621	1197
Relationship status			
Cohabiting partner	1232 (69)	449 (73)	777 (67)
Single / no current partner	554 (31)	163 (27)	387 (33)
Total	1786	612	1164
Highest qualification			
Postgraduate	423 (23)	136 (22)	287 (24)
Degree	456 (25)	171 (28)	285 (24)
Further education	515 (29)	180 (29)	335 (28)
Highers	170 (9)	51 (8)	119 (10)
Standard/O grades	172 (10)	48 (8)	123 (10)
Other	39 (2)	14 (2)	25 (3)
None	29 (2)	12 (2)	17 (1)
Total	1804	612	1191
Occupational group			
Managerial	86 (5)	28 (5)	58 (5)
Professional	399 (23)	111 (19)	288 (25)
Associate professional & technical	508 (29)	235 (39)	273 (24)
Administrative & secretarial	556 (32)	148 (25)	408 (36)
Skilled trades	21 (1)	15 (2.5)	6 (1)
Caring/leisure/other services	147 (8)	39 (6.5)	108 (9)
Sales/customer services	2 (0.1)	0	2 (0.2)
Process/plant/machine operators	12 (1)	12 (2)	0
Elementary	12 (1)	9 (1)	3 (0.3)
Total	1743	597	1146
Employment status			
Full-time employment	1584 (88)	603 (97)	981 (83)
Part-time employment	224 (12)	17 (3)	207 (17)
Total	1808	620	1188
Pain status			
No pain	857 (53)	330 (58)	527 (50)
Acute pain	126 (8)	45 (8)	81 (8)
Chronic pain	642 (40)	194 (34)	448 (42)
Total	1625	569	1056

### Logistic regression modelling for males and females

The acute pain regression model identified no significant predictors of acute pain among men and women and is not reported. The model for chronic pain was significant, for both sexes (Table 2). Women were 64% more likely to report chronic pain than men (inverted OR: 0.60; 95% confidence interval: 0.44–0.81). Among men, age, highest educational qualification, general health, fair pay and distributive justice were associated with chronic pain. Men with higher perceptions of fair pay (compared to lower perceptions of fair pay) were 25% more likely to report chronic pain (OR: 1.25; 95% confidence interval: 1.03–1.53). Men with higher perceptions of distributive justice were less likely to report chronic pain than those with lower perceptions of distributive justice (OR: 0.76; 95% confidence interval: 0.59–0.98). Compared to younger male employees (16–34 years), males aged 35–44 were almost three times as likely to report chronic pain (OR: 3.00; 95% confidence interval: 1.25–7.21), while those over 45 years of age were almost four times as likely to report chronic pain (OR: 3.89; 95% confidence interval: 1.61–9.41). Male employees with a postgraduate qualification (OR: 3.06; 95% confidence interval: 1.24–7.57), a degree (OR: 2.54; 95% confidence interval: 1.09–5.91) or a further education qualification (OR: 3.58; 95% confidence interval: 1.57–8.16) were up to three times as likely to report chronic pain than employees with only school level or no qualifications. In terms of self-reported general health, for every additional one-unit increase in a man’s health, he was 3% less likely to report chronic pain than those who reported poorer health (OR: 0.97; 95% confidence interval: 0.96–0.98).

**Table 2.** Logistic regression analysis for chronic pain.

	All^a^			Men^a^			Women^a^		
	OR^b^	CI^c^		OR	CI		OR	CI	
Gender	0.60**	0.44	0.81						
Age_group 35–44	1.92**	1.27	2.91	3.00**	1.25	7.21	1.69*	1.03	2.73
Age_group 45+	3.28**	2.15	5.01	3.89**	1.61	9.41	3.38**	2.05	5.59
Partner	1.24	0.93	1.67	1.53	0.87	2.68	1.17	0.82	1.68
SOC^d^_2 Professionals	0.74	0.41	1.34	0.68	0.19	2.46	0.73	0.37	1.44
SOC_3 Associate prof/tech	1.15	0.74	1.79	0.93	0.40	2.11	1.28	0.74	2.22
SOC_4 Admin/secretarial	1.55*	0.99	2.44	1.25	0.56	2.77	1.76*	1.01	3.06
Gross weekly earnings	1.00	1.00	1.00	1.00	1.00	1.00	1.00	1.00	1.00
Full-time employment	1.44	0.94	2.20	1.03	0.13	8.15	1.38	0.89	2.15
Postgraduate qualification	1.92*	1.13	3.26	3.06*	1.24	7.57	1.63	0.81	3.28
Degree	1.98**	1.19	3.30	2.54*	1.09	5.91	1.80	0.92	3.54
FE^e^ qualification	1.99**	1.23	3.21	3.58**	1.57	8.16	1.56	0.84	2.89
Highers	1.36	0.75	2.48	1.68	0.59	4.76	1.24	0.58	2.64
General health	0.98**	0.97	0.98	0.97**	0.96	0.98	0.98**	0.97	0.99
Smoking	1.27	0.86	1.87	0.93	0.47	1.84	1.65*	1.00	2.75
Total alcohol	0.85	0.68	1.06	0.98	0.65	1.47	0.83	0.62	1.10
Total alcohol^2^	1.01	0.99	1.03	1.00	0.97	1.04	1.01	0.98	1.04
Exercise 1–11 days per month	0.78	0.49	1.24	0.84	0.31	2.27	0.78	0.46	1.35
Exercise 12–19 days per month	0.58*	0.34	0.98	0.81	0.27	2.44	0.5*	0.27	0.93
Exercise 20+ days per month	0.56*	0.34	0.91	0.54	0.20	1.46	0.59	0.33	1.05
Fair_pay	1.05	0.94	1.17	1.25*	1.03	1.53	0.98	0.85	1.12
Distributive justice	0.88*	0.77	0.99	0.76*	0.59	0.98	0.93	0.81	1.08
Procedural justice	1.05	0.94	1.17	1.06	0.86	1.31	1.07	0.94	1.22
Interpersonal justice	1.00	0.91	1.09	0.98	0.83	1.15	1.03	0.92	1.14
Total satisfaction	1.00	0.99	1.01	0.99	0.98	1.00	1.00	0.99	1.01
Total stress	1.01**	1.01	1.02	1.01	0.99	1.02	1.02**	1.01	1.03
Constant	0.57	0.12	2.69	0.89	0.04	22.69	0.18	0.03	1.55
	Log likelihood -679.42846			Log likelihood -220.76341			Log likelihood -443.8769		
	Prob > Chi2 0.0000			Prob > Chi2 0.0000			Prob > Chi2 0.0000		
	Pseudo R2 0.1366			Pseudo R2 0.183			Pseudo R2 0.134		
	*N* 1156			*N* 411			*N* 745		

**significant at 0.01%, *significant at 0.05%.

a Model significant at 0.001%.

b Odds ratio.

c Confidence interval.

d Standard occupational classification.

e Further Education.

For women, age, administrative/secretarial occupation, general health, smoking, exercise and stress were associated with chronic pain but no organisational justice variables were significant predictors. Like the men, older women, particularly those aged 45 years and over, were over three times as likely to report chronic pain compared to their younger female counterparts (OR: 3.38; 95% confidence interval: 2.05–5.59) (Table 2). Female employees in administrative/secretarial occupations were 76% more likely to report experiencing chronic pain than those in other occupations (OR: 1.76; 95% confidence interval: 1.01–3.06). In terms of general health status, like men, women who reported better health were less likely to report chronic pain than those who reported poorer health (OR: 0.98; 95% confidence interval: 0.97–0.99), and women who smoked were 65% more likely to report chronic pain compared to female non-smokers. (OR: 1.65, 95% confidence interval: 1.00–2.75). Additionally, for each one-unit increase in a woman’s total stress score, they were 2% more likely to report chronic pain (OR: 1.02; 95% confidence interval: 1.01–1.03). Finally, women who exercised for between 12 and 19 days per month were only half as likely to report chronic pain compared to those who reported less exercise (OR: 0.5; 95% confidence interval: 0.27–0.93).

## Discussion

This study examined the association of organisational justice with acute and chronic pain among male and female employees. Work, health and demographic variables were controlled for in the analysis, and job satisfaction and psychological stress were included.

Among men only, perceptions of fair pay and distributive justice were unique predictors of chronic pain. Specifically, men with a higher perception of fair pay compared to a lower perception were more likely to report chronic pain, as were men who reported a low level of distributive justice. We are uncertain about the meaning of the positive interaction between fair pay and pain among men. It is possible this is a chance finding but we suggest the finding aligns with research showing that pay equity is an important concern of male employees, as a symbol of how their work is evaluated and a measure of their status at work (Khoreva and Tenhiala, 2016; Tata, 2000). Although not found in this study, research indicates women may have other priorities linked to procedural justice to achieve desired outcomes through fair decision-making processes at work (Khoreva and Tenhiala, 2016). This interpretation, however, remains speculative until further research is conducted examining the implications of perceptions of fair pay among employees with chronic pain.

Whilst satisfied that they were paid fairly, the findings indicated that male employees with chronic pain had lower perceptions of distributive justice in relation to rewards such as recognition, promotion or benefits. It is possible that, while some outcomes may be set, in terms of pay, employees with chronic pain perceive that other, more flexible outcomes and opportunities are withheld from them or are out of their reach due to their pain condition, such as the opportunity for promotion (Beatty, 2012). This interpretation is consistent with research showing that individuals with chronic illness can feel under threat of being stigmatised at work (McGonagle and Barnes-Farrell, 2013), in relation to productivity and the ability to maintain regular working hours (Beatty and Joffe, 2006). It is also possible that, for the employee with chronic pain, weaker perceptions of distributive justice reflect issues associated with work accommodations such as flexible working hours or adjustment to type of work or working hours that are needed to manage pain at work (Grant et al., 2019). However, further investigation is needed to understand the nature of the perceptions of distributive justice in this group.

The finding with respect to distributive justice supports that of previous research (Freimann et al., 2016; Pekkarinen et al., 2013), including those of Saastamoinen et al. (2009) who found organisational justice was associated with chronic pain among male but not female employees. Although organisational justice was unrelated to chronic pain among females, it is possible that men and women experience justice-related issues differently from each other. For example, research suggests that organisational justice is associated with different work-related outcomes for male and female employees in general (Jepsen and Rodwell, 2010; Ramamoorthy and Flood, 2004). This is worthy of future investigation among employees with pain.

In other findings, consistent with previous research, women, older employees and those with poorer health were more likely to report chronic pain among men and women (Fillingim, 2017; Saastamoinen et al., 2006). Among women only, working in administrative or clerical occupations, higher self-reported stress and smoking behaviour were predictors of self-reported chronic pain, while moderate exercise (exercising for between 12 and 19 days per month) was associated with less likelihood of reporting chronic pain. These findings support the patterning of previous research (Ditre et al., 2011; Landmark et al., 2011; Widanarko et al., 2012). However, adding to the mixed results of previous work (McNamara et al., 2017) a higher rather than a lower level of education was associated with an increased likelihood of reporting chronic pain among male employees.

The results contribute to research on psychosocial and behavioural factors associated with pain among employees, particularly with respect to the growing body of research indicating the importance of organisational justice to employees with chronic pain. These perceptions of work have potential implications for clinical decision -makers who recognise work attitudes as potential ‘blue flags’ to be addressed as part of treatment to support the employee with chronic pain (Shaw et al., 2009). There are also implications for the employer, in relation to their processes for supporting the employee with chronic pain to offset or minimise the potential negative repercussions of weak organisational justice beliefs in relation to, for example, job satisfaction, commitment and work performance (Van Dijke and De Cremer, 2016). Evidence suggests training employers in organisational justice techniques can improve perceptions of fairness and occupational functioning among employees (Nakamura et al., 2016).

A strength of the study is that we sought to recruit all employees, regardless of pain status, thus reducing the potential for bias had we actively recruited only employees with chronic pain. A study limitation is that causality cannot be established from the data. Future research should examine the bi-directional relationship between organisational justice and chronic pain to determine the direction of intervention work. Additionally, caution is needed with the generalisation of the findings, as the sample were predominantly middle-aged, co-habiting, educated females from a range of professions, among whom musculoskeletal pain was more commonly reported. Although there was an approximate 10% response rate, there was a large variance in all key phenomena that enabled these analyses to be performed with good internal validity for this sample. A third limitation is that, while single item measures can provide useful information in the context of organisational research (Fisher et al., 2016), multiple item measures tend to have stronger psychometric properties and should be considered in future research.

Notwithstanding these limitations, this is the first study to find that fair pay and distributive justice are both unique predictors of chronic pain. Future research is needed to investigate these findings further and consider gender differences in justice-related reasoning within the workplace, and the implications of justice-related reasoning for the employee with pain.

## References

[bibr1-1359105320967423] Ariens GA van Mechelen W Bongers PM , et al. (2001) Psychosocial risk factors for neck pain: A systematic review. American Journal of Industrial Medicine 39: 180–93. 10.1002/1097-0274(200102)39:2<180::aid-ajim1005>3.0.co;2-# 11170160

[bibr2-1359105320967423] Beatty JE (2012) Career barriers experienced by people with chronic illness: A U.S. study. Employee Responsibility Rights Journal 2: 91–110.

[bibr3-1359105320967423] Beatty JE Joffe R (2006) An overlooked dimension of diversity: The career effects of chronic illness. Organisational Dynamics 35: 182–195.

[bibr4-1359105320967423] Bonzini M Bertu L Veronesi G , et al. (2015) Is musculoskeletal pain a consequence or a cause of occupational stress? A longitudinal study. International Archives of Occupational and Environmental Health 88: 607–612. 25261316 10.1007/s00420-014-0982-1 PMC4437793

[bibr5-1359105320967423] Bjorner JB Pejtersen JH (2010) Evaluating construct validity of the second version of the Copenhagen Psychosocial Questionnaire through analysis of differential item functioning and differential item effect. Scandinavian Journal of Public Health 38: 90–105. 21172775 10.1177/1403494809352533

[bibr6-1359105320967423] Bowling A (2005) Just one question: if one question works, why ask several? Journal of Epidemiology and Community Health 59: 342–345. 15831678 10.1136/jech.2004.021204 PMC1733095

[bibr7-1359105320967423] Breivik H Eisenberg E O’Brien T , et al. (2013) The individual and societal burden of chronic pain in Europe: The case for strategic prioritisation and action to improve knowledge and availability of appropriate care. BMC Public Health 13: 1229. 24365383 10.1186/1471-2458-13-1229 PMC3878786

[bibr8-1359105320967423] Buruck G Tomaschek A Wendsche J , et al. (2019) Psychosocial areas of worklife and chronic low back pain: a systematic review and meta-analysis. BMC Musculoskeletal Disorders 20: 480. 31653249 10.1186/s12891-019-2826-3 PMC6814972

[bibr9-1359105320967423] Colquitt JA Shaw JC (2005) How should organizational justice be measured? In: Greenberg J Colquitt JA (eds) The Handbook of Organizational Justice. Mahwah, NJ: Erlbaum, pp.113–152.

[bibr10-1359105320967423] Ditre JW Brandon TH Zale EL , et al. (2011) Pain, nicotine, and smoking: Research findings and mechanistic considerations. Psychological Bulletin 137: 1065–1093. 21967450 10.1037/a0025544 PMC3202023

[bibr11-1359105320967423] Eib C Bernhard-Oettel C Magnusson Hanson LL , et al. (2018) Organizational justice and health: studying mental preoccupation with work and social support as mediators for lagged and reversed relationships. Journal of Occupational Health Psychology 23: 553–567. 29504778 10.1037/ocp0000115

[bibr12-1359105320967423] Elliot AM Burton CD Hannaford PC (2014) Resilience does matter: Evidence from a 10-year cohort record linkage study. British Medical Journal Open 4: e003917. 10.1136/bmjopen-2013-003917 PMC3902361 24430878

[bibr13-1359105320967423] Fayaz A Croft P Langford RM , et al. (2016). Prevalence of chronic pain in the UK: A systematic review and meta-analysis of population studies. BMJ Open 6: e010364. 10.1136/bmjopen-2015-010364 PMC4932255 27324708

[bibr14-1359105320967423] Fillingim RB (2017) Individual differences in pain: Understanding the mosaic that makes pain personal. Pain 158 (suppl 1): S11–S18. 27902569 10.1097/j.pain.0000000000000775 PMC5350021

[bibr15-1359105320967423] Fisher GG Matthews RA Gibbons AM (2016) Developing and investigating the use of single-item measures in organizational research. Journal of Occupational Health Psychology 21: 3–23. 25894198 10.1037/a0039139

[bibr16-1359105320967423] Freimann T Pääsuke M Merisalu E (2016) Work-related psychosocial risk factors and mental health problems associated with musculoskeletal pain in nurses: A cross sectional study. Pain Research and Management 3: 9361016. 10.1155/2016/9361016 PMC5112316 27885319

[bibr17-1359105320967423] Gatchel RJ Peng YB Peters ML , et al. (2007) The biopsychosocial approach to chronic pain: Scientific advances and future directions. Psychological Bulletin 133(4): 581–624. 17592957 10.1037/0033-2909.133.4.581

[bibr18-1359105320967423] Grant M Rees S Underwood M , et al. (2019) Obstacles to returning to work with chronic pain: In-depth interviews with people who are off work due to chronic pain and employers. BMC Musculoskeletal Disorders 20: 486. 31656184 10.1186/s12891-019-2877-5 PMC6815386

[bibr19-1359105320967423] Heponiemi T Elovainio M Kouvonen A , et al. (2013) Can organizational justice mitigate the negative effects of shift work and fixed-term employment? European Journal of Work and Organizational Psychology 22: 194–202.

[bibr20-1359105320967423] Herr RM Almer C Bosle C , et al. (2020) Associations of changes in organisational justice with job attitudes and health -Findings from a prospective study using a matching-based difference-in-difference approach. International Journal of Behavioral Medicine, 27: 119–135. 31879857 10.1007/s12529-019-09841-z

[bibr21-1359105320967423] Herr RM Bosch JA Loerbroks A , et al. (2015) Three job stress models and their relationship with musculoskeletal pain in blue-and white-collar workers. Journal of Psychosomatic Research 79(5): 340–347. 26526306 10.1016/j.jpsychores.2015.08.001

[bibr22-1359105320967423] International Association for the Study of Pain. (1986) Classification of chronic pain. Pain Suppl 3: S1–226. 3461421

[bibr23-1359105320967423] Jepsen D Rodwell J (2010) Female perceptions of organisational justice. Gender, Work and Organisation 19: 723–740.

[bibr24-1359105320967423] Jordan JS Turner BA (2008) The feasibility of single-item measures for organizational justice. Measurement in Physical Education and Exercise Science 12: 237–257.

[bibr25-1359105320967423] Khoreva V Tenhiälä A (2016) Gender differences in reactions to injustice. Journal of Managerial Psychology 31: 790–804.

[bibr26-1359105320967423] Kompier MAJ Taris TW (2011) Understanding the causal relations between psychosocial factors at work and health: A circular process. Scandinavian Journal of Work Environment & Health 37: 259–262. 10.5271/sjweh.3172 21643622

[bibr27-1359105320967423] Landmark T Romundstad P Borchgrevink PC , et al. (2011) Associations between recreational exercise and chronic pain in the general population: Evidence from the HUNT 3 study. Pain 152: 2241–2247. 21601986 10.1016/j.pain.2011.04.029

[bibr28-1359105320967423] Lang J Ochsmann E Kraus T , et al. (2012) Psychosocial work stressors as antecedents of musculoskeletal problems: A systematic review and meta-analysis of stability-adjusted longitudinal studies. Social Science and Medicine 75: 1163–1174. 22682663 10.1016/j.socscimed.2012.04.015

[bibr29-1359105320967423] Manville C El Akremi A Niezborala M , et al. (2016) Injustice hurts, literally: The role of sleep and emotional exhaustion in the relationship between organizational justice and musculoskeletal disorders. Human Relations 69: 1315–1339.

[bibr30-1359105320967423] Matta FK Scott B Colquitt J , et al. (2017) Is consistently unfair better than sporadically fair? An investigation of justice variability and stress. Academy of Management Journal 60: 743–770.

[bibr31-1359105320967423] McGonagle A Barnes-Farrell J (2013) Chronic illness in the workplace: Stigma, identity threat, and strain. Stress & Health 30: 310–321. 23955842 10.1002/smi.2518

[bibr32-1359105320967423] McNamara CL Balaj M Thomson KH , et al. (2017) The socioeconomic distribution of non-communicable diseases in Europe: Findings from the European Social Survey (2014) special module on the social determinants of health. European Journal of Public Health 27: 22–26. 10.1093/eurpub/ckw222 28355638

[bibr33-1359105320967423] Milton K Bull FC Bauman A (2011) Reliability and validity testing of a single-item physical activity measure. British Journal of Sports Medicine 45: 203–208. 20484314 10.1136/bjsm.2009.068395

[bibr34-1359105320967423] Nakamura S Somemura H Sasaki N , et al. (2016) Effect of management training in organizational justice: a randomized controlled trial. Industrial Health 54: 263–271. 26860786 10.2486/indhealth.2015-0164 PMC4939869

[bibr35-1359105320967423] Ndjaboue R Brisson C Vezina M (2012) Organisational justice and mental health: A systematic review of prospective studies. Occupational and Environmental Medicine 69: 694–700. 22693265 10.1136/oemed-2011-100595

[bibr36-1359105320967423] Nicholas M Vlaeyen JWS Rief W , et al. (2019) The IASP taskforce for the classification of chronic pain. The IASP classification of chronic pain for ICD-11: Chronic primary pain. Pain 160: 28–37. 30586068 10.1097/j.pain.0000000000001390

[bibr37-1359105320967423] Pain Alliance Europe (2018) Survey on chronic pain and your work life. A survey in 14 European countries.

[bibr38-1359105320967423] Patel AS Farquharson R Carroll D , et al. (2012) The impact and burden of chronic pain in the workplace: A qualitative systematic review. Pain Practice 12: 578–589. 22462774 10.1111/j.1533-2500.2012.00547.x

[bibr39-1359105320967423] Pejtersen JH Kristensen TS Borg V , et al. (2010) The second version of the Copenhagen psychosocial questionnaire. Scandinavian Journal of Public Health 38: 8–24. 21172767 10.1177/1403494809349858

[bibr40-1359105320967423] Pekkarinen L Elovainio M Sinervo T , et al. (2013) Job demands and musculoskeletal symptoms among female geriatric nurses: The moderating role of psychosocial resources. Journal of Occupational Health and Psychology 18: 211–219. 10.1037/a0031801 23458058

[bibr41-1359105320967423] Philips CJ (2009) The cost and burden of chronic pain. Reviews in Pain 3: 2–5. 10.1177/204946370900300102 PMC4590036 26526940

[bibr42-1359105320967423] Ramamoorthy N Flood PC (2004) Gender and employee attitudes: The role of organizational justice perceptions. British Journal of Management 15: 247–258.

[bibr43-1359105320967423] Robbins JM Ford MT Tetrick LE (2012) Perceived unfairness and employee health: A meta-analytic integration. Journal of Applied Psychology 97: 235–272. 10.1037/a0025408 21928872

[bibr44-1359105320967423] Rodrigues MSA Liete RDV Lelis CM , et al. (2017) Differences in ergonomic and workstation factors between computer office workers with and without reported musculoskeletal pain. Work 5: 1–10. 10.3233/WOR-172582 28826196

[bibr45-1359105320967423] Saastamoinen P Laaksonen M Leino-Arjas P , et al. (2009) Psychosocial risk factors of pain among employees. European Journal of Pain 13: 102–108. 18440254 10.1016/j.ejpain.2008.03.006

[bibr46-1359105320967423] Saastamoinen P Leino-Arjas P Laaksonen M , et al. (2006) Pain and health related functioning among employees. Journal of Epidemiology and Community Health 60: 793–798. 16905725 10.1136/jech.2005.043976 PMC2566029

[bibr47-1359105320967423] Shaw WS van der Windt DA Main CJ , et al. (2009) Early patient screening and intervention to address individual-level occupational factors (“Blue flags”) in back pain disability. Journal of Occupational Rehabilitation 19: 64–80. 19082875 10.1007/s10926-008-9159-7

[bibr48-1359105320967423] Skillgate E Vingard E Josephson M , et al. (2009) Is smoking and alcohol consumption associated with long-term sick leave due to unspecific back or neck pain among employees in the public sector? Results of a three-year follow-up cohort study. Journal of Rehabilitation Medicine 41: 550–556. 19543666 10.2340/16501977-0370

[bibr49-1359105320967423] Soer R De Vries HJ Brouwer S , et al. (2012) Do workers with chronic nonspecific musculoskeletal pain, with and without sick leave, have lower functional capacity compared with healthy workers? Archives of Physical Medicine and Rehabilitation 93: 2216–2222. 22772082 10.1016/j.apmr.2012.06.023

[bibr50-1359105320967423] Tata J (2000) Influence of role and gender on the use of distributive versus procedural justice principles. Journal of Psychology 134: 261–268. 10.1080/00223980009600866 10907704

[bibr51-1359105320967423] Tenhiälä A Linna A Von Bonsdorff M , et al. (2013) Organizational justice, sickness absence and employee age. Journal of Managerial Psychology 28: 805–825.

[bibr52-1359105320967423] Thorsen SV Bjorner JB (2010) Reliability of the Copenhagen psychosocial questionnaire. Scandinavian Journal of Public Health 38: 8–24. 21172768 10.1177/1403494809349859

[bibr53-1359105320967423] van Dijke MH De Cremer D (2016) Justice in the Work Setting. In: Sabbagh C Schmitt M (eds) Handbook of Social Justice Theory and Research. New York, NY: Springer, pp. 315–332.

[bibr54-1359105320967423] White M Wagner S Schultz IZ , et al. (2013) Modiﬁable workplace risk factors contributing to workplace absence across health conditions: A stakeholder-centred best evidence synthesis of systematic reviews. Work: A Journal of Prevention, Assessment & Rehabilitation 45: 1–12. 10.3233/WOR-131628 23531590

[bibr55-1359105320967423] Widanarko B Legg S Stevenson M , et al. (2012) Gender differences in work related risk factors associated with low back symptoms. Ergonomics 55: 327–342. 22409170 10.1080/00140139.2011.642410

